# Family cancer history and smoking habit associated with sarcoma in a Japanese population study

**DOI:** 10.1038/s41598-022-21500-0

**Published:** 2022-10-12

**Authors:** Yoshihiro Araki, Norio Yamamoto, Yoshikazu Tanzawa, Takahiro Higashi, Aya Kuchiba, Katsuhiro Hayashi, Akihiko Takeuchi, Shinji Miwa, Kentaro Igarashi, Makoto Endo, Eisuke Kobayashi, Hiroyuki Tsuchiya, Akira Kawai

**Affiliations:** 1 Division of Musculoskeletal Oncology, National Cancer Center Hospital, 5-1-1 Tsukiji, Chuo-ku, Tokyo, 104-0045 Japan; 2 Department of Orthopaedic Surgery, Graduate School of Medical Sciences, Kanazawa University Hospital, Kanazawa, Japan; 3 Department of Orthopaedics, Tokai University School of Medicine, Yokohama, Japan; 4 Institute of Cancer Control, National Cancer Center Hospital, Tokyo, Japan; 5 Graduate School of Health Innovation, Kanagawa University of Human Services, Kanagawa, Japan; 6 Department of Orthopaedic Surgery, Kyushu University, Fukuoka, Japan

**Keywords:** Genetics, Health care, Oncology, Risk factors

## Abstract

Sarcoma is a rare cancer, and little is known about the etiology, lifestyle epidemiology, and actual circumstances of treatment in hospitals in Japan. Understanding these issues is essential for the effective prevention and treatment of sarcoma. We therefore investigated the incidence of a personal and family cancer history in a total of 1320 sarcoma patients at the National Cancer Center Hospital. In addition, obesity, hypertension, dyslipidemia, diabetes mellitus, drinking, smoking, age and sex were compared in a descriptive study of 1159 of these sarcoma patients who were ≥ 20 years of age, and 7738 controls derived from the National Health and Nutrition Examination Survey in Japan. A total of 8% of sarcoma patients had a personal history of another cancer, and 30% of soft tissue sarcoma patients had a family cancer history in a first-degree relative (malignant peripheral nerve sheath tumor, 52%; leiomyosarcoma, 46%). A smoking habit was associated with the development of sarcoma (odds ratio [OR], 2.05; 95% confidence interval, 1.78–2.37; *p* < 0.01). According to the histology, the ORs for undifferentiated pleomorphic sarcoma (UPS) of bone, UPS of soft tissue, and liposarcoma were 5.71, 3.04, and 2.92, respectively. A family cancer history may be associated with certain soft tissue sarcomas, and a smoking habit was significantly associated with the development of sarcomas; however, further studies are necessary.

## Introduction

Sarcoma is a rare cancer, and the annual incidence of soft tissue sarcoma is approximately 50 cases per 1 million population, while that of bone sarcoma is approximately 5 cases per 1 million population, and there are more than 50 distinct histological subtypes of sarcoma in the 2013 World Health Organization (WHO) classification^1^. The rarity of the disease and the diversity of their subtypes make it difficult to investigate the factors in personal and family history of cancer that are possibly associated with novel genetic disorders, and in addition, to examine the risk factors for sarcoma, such as lifestyle habits and lifestyle-related diseases; thus, the knowledge about its etiology, lifestyle epidemiology, and actual circumstances of treatment in hospitals in Japan is limited^2,3^. Understanding these issues is essential for the management of these diseases, and providing standard treatment for sarcoma; thus, its elucidation is urgently demanded.

A personal or family cancer history increases the risk of breast, colorectal, gastric, and bladder cancer^4–9^, which includes the genetic disorders such as *BRCA1/2*, *BRAF*, *KRAS*, and *p53*. In the previous studies, 30–40% of sarcoma patients had a first-degree relative with a history of cancer (i.e., breast cancer, lung cancer); however, the histological types of sarcoma are not specified due to the relatively small study population^10–12^. Some syndromes are associated with both personal or family history of cancer and sarcoma. Li–Fraumeni syndrome is well known to be associated with a personal or family history of breast cancer, and the development of certain subtypes of sarcoma, including rhabdomyosarcoma, liposarcoma, leiomyosarcoma, and osteosarcoma, and this syndrome is caused by a *p53* gene mutation^13,14^. Lynch syndrome is also associated with a personal or family history of colorectal cancer, in addition to the occurrence of certain types of sarcoma, such as undifferentiated pleomorphic sarcoma (UPS) and bone tumor, and this syndrome occurs because of microsatellite instability and/or the deletion of mismatch repair genes of *MLH1*, *MSH2*, *MSH6*, and *PMS2*^15,16^.

It is also essential to examine the association of lifestyle habits (drinking and smoking) and lifestyle-related diseases (obesity, diabetes mellitus [DM], hypertension [HT], dyslipidemia [DL]) with sarcoma, as these factors have previously been shown to be involved in the development of cancers through a variety of mechanisms^17–34^. However, the association between these factors and sarcomas has not been sufficiently established. A cohort study revealed a relationship between smoking habit and the development of soft tissue sarcoma approximately 3 decades ago^35^, and a few case–control studies with a relatively small study populations reported that smoking habit was associated with the development of sarcoma with ORs of 1.8–2.7^11,36^. These studies included all types of sarcoma together and little is known about the histological subtypes of sarcoma associated with smoking.

We therefore investigated the personal and family cancer history in bone and soft tissue sarcoma patients. In addition, we investigated the lifestyle habits and lifestyle-related diseases of a population of bone and soft tissue sarcoma patients and compared them to those of healthy controls based on National Health and Nutrition Examination Survey (NHNES) data^37^.

## Results

### Demographic data of cases and controls

The sample of sarcoma patients included more females than males (56% vs. 44%). The median age was 49 years (range 1–97 years). Among sarcoma patients, 161 (12%) patients were < 20 years of age, 274 (21%) had bone sarcoma while the remaining 1046 (79%) had soft tissue sarcoma. The 1159 patients of ≥ 20 years of age (male, n = 647; female, n = 512; a median age, 54 years [range 20–97 years]) included 193 patients with bone sarcoma and 966 patients with soft tissue sarcoma.

### Personal cancer history in sarcoma patients

In total, 8% of the patients (108/1320) with sarcoma of the bone or soft tissue had a personal cancer history. The rate of a personal cancer history among the bone sarcoma patients was 5% (14/274), while that among the soft tissue sarcoma patients was 9% (94/1046) (Table 1). No personal cancer history was observed in > 10% of patients with any type of bone sarcoma, while patients with angiosarcoma (38%) and UPS (17%) had a personal history of another cancer (Supplementary Table 1). The most common types of historical cancer in soft tissue sarcoma patients were breast (16%), colorectum (11%), and lung cancer (10%) (Table 2).

**Table 1:** Personal and family history of cancer in patients with sarcoma.

	Cases	Family history of cancer			Personal cancer history (%)
		Up to third-degree relatives (%)	Up to second-degree relatives (%)	First-degree relatives (%)	
**Bone and soft tissue sarcoma**	1320	730 (55%)	651 (49%)	367 (28%)	108 (8%)
**Bone sarcoma**	274	142 (52%)	127 (46%)	56 (20%)	14 (5%)
Male	159	80 (50%)	72 (45%)	34 (21%)	9 (6%)
Female	115	62 (54%)	55 (48%)	22 (19%)	5 (4%)
**Soft tissue sarcoma**	1046	578 (55%)	524 (50%)	311 (30%)	94 (9%)
Male	580	306 (53%)	279 (48%)	160 (28%)	40 (7%)
Female	466	272 (58%)	245 (53%)	151 (32%)	54 (12%)

**Table 2:** Other types of cancer in sarcoma patients.

Types of cancer	Bone sarcoma (n = 274)	Soft tissue sarcoma (n = 1046)
Gastric cancer	3	5
Lung cancer	2	9
Colorectal cancer	3	10
Breast cancer	0	15
Thyroid cancer	0	7
Ovarian cancer	0	6
Uterine cancer	0	5
Prostate cancer	2	8
Esophageal cancer	0	2
Renal cell cancer	0	2
Bladder cancer	2	3
Others	3	23
Total	14 (5%)	94 (9%)

### Family cancer history in sarcoma patients

The family history of cancer among the patients with sarcoma of the bone or soft tissue was as follows: up to first-degree, n = 367 (28%); up to second-degree, n = 651 (49%); and up to third-degree relatives, n = 730 (55%) (Table 1). The rates of patients with a family history of cancer up to first-, second-, and third-degree relatives were 20% (56/274), 46% (127/274), and 52% (142/274), respectively, in bone sarcoma patients, and 30% (311/1046), 50% (524/1046), and 55% (578/1046), respectively, in soft tissue sarcoma patients (Table 1). According to histological type, patients with malignant peripheral nerve sheath tumor (MPNST) (52% [13/25]), leiomyosarcoma (46% [26/57]), extraskeletal myxoid chondrosarcoma (EMC) (42% [5/12]), chordoma (41% [7/17]), and synovial sarcoma (39% [21/54]) had relatively high rates of family cancer history up to first-degree relatives (Supplementary Table 1). Among bone and soft tissue sarcoma patients, the most common types of cancer up to first-degree relatives were gastric (124/1320), lung (81/1320), and colorectal cancer (73/1320) (Table 3).

**Table 3:** Types of cancer in first-degree relatives of sarcoma patients.

Types of cancer	Bone sarcoma (n = 274)	Soft tissue sarcoma (n = 1046)
Gastric cancer	17	107
Lung cancer	10	71
Colorectal cancer	11	62
Breast cancer	9	29
Hepatic cell cancer	4	33
Uterine cancer	5	29
Pancreatic cancer	1	29
Prostate cancer	2	16
Esophageal cancer	2	15
Renal cell cancer	1	7
Bladder cancer	2	8
Others	16	77
Total	56 (20%)	311 (30%)

### Comparison between sarcoma patients and the normal population.

A total of 8897 subjects (1159 cases and 7738 controls) were analyzed (Table 4). Twenty-seven percent of sarcoma patients and 20% of controls were 20–39 years of age (*p* < 0.01). Male patients accounted for 56% of sarcoma patients and 47% of controls (*p* < 0.01). Thirty-six percent of sarcoma patients and 19% of controls had a smoking habit (*p* < 0.01). Six percent of sarcoma patients and 4% of controls had DM (*p* < 0.01). Other comorbidities (alcohol drinking habit, hypertension, obesity, and DL) did not reach the pre-specified significance level. The highest OR was 2.05 (95% CI 1.78–2.37, *p* < 0.01) for sarcoma patients with a smoking habit.

**Table 4:** Sample characteristics and univariate and multivariate analyses of risk factors for sarcoma.

	Cases (n = 1159)	Controls (n = 7738)	Univariate			Multivariate		
			OR	95% CI	*P*-value	OR	95% CI	*P*-value
**Age [years old]**								
20–39	316 (27%)	1540 (20%)	1.51	1.31–1.74	< 0.01	1.71	1.44–2.02	< 0.01
40–59	377 (33%)	2352 (30%)	1.1	0.96–1.26	0.14	1.26	1.08–1.47	< 0.01
60–	466 (40%)	3846 (50%)	0.68	0.59–0.77	< 0.01	Ref		
**Sex**								
Male	647 (56%)	3617 (47%)	1.44	1.27–1.63	< 0.01	1.09	0.95–1.26	0.20
Female	512 (44%)	4121 (53%)	Ref			Ref		
**Smoking habit**								
(+)	414 (36%)	1497 (19%)	2.31	2.02–2.65	< 0.01	2.05	1.78–2.37	< 0.01
(−)	745 (64%)	6241 (81%)	Ref			Ref		
**Alcohol drinking habit**								
(+)	303 (26%)	1566 (20%)	1.40	1.21–1.61	< 0.01	1.15	0.98–1.34	0.10
(−)	856 (74%)	6172 (80%)	Ref			Ref		
**Obesity**								
(+)	255 (22%)	1436 (19%)	1.24	1.06–1.44	< 0.01	1.22	1.05–1.43	0.02
(−)	904 (78%)	6302 (81%)	Ref			Ref		
**DM**								
(+)	70 (6%)	290 (4%)	1.65	1.24–2.17	< 0.01	1.87	1.40–2.51	< 0.01
(−)	1089 (94%)	7448 (96%)	Ref			Ref		
**HT**								
(+)	183 (16%)	1200 (16%)	1.02	0.86–1.21	0.79	1.23	1.01–1.51	0.04
(−)	976 (84%)	6538 (84%)	Ref			Ref		
**DL**								
(+)	79 (7%)	706 (9%)	0.72	0.56–0.93	< 0.01	0.72	0.55–0.94	0.02
(−)	1080 (93%)	7032 (91%)	Ref			Ref		

*Ref* reference, *DM* Diabetes mellitus, *HT* Hypertention, *DL* Dyslipidemia, *OR* Odds ratio, *CI* Confidence interval.

The smoking rates according to histological type are described in Supplementary Table 2. Among them, the smoking rate was 320/914 (35%) in patients with high-grade sarcoma, and 94/245 (38%) in patients with low or intermediate-grade sarcoma (*p* = 0.33). Furthermore, the OR for soft tissue sarcoma was 2.16 (95% CI 1.85–2.52, *p* < 0.01), and was slightly higher than the OR for bone sarcoma (OR: 1.55, 95% CI 1.11–2.16, *p* < 0.01), although there were no significant differences (*p* = 0.37) (Table 5).

**Table 5:** Smoking rates in sarcoma patients of ≥ 20 years of age according to histological grade and disease origin.

	Controls	Cases				
		Sarcoma	High-grade sarcoma	Low-grade and intermediate sarcoma	Bone sarcoma	Soft tissue sarcoma
**N**	7738	1159	914	245	193	966
**Smoking habit (%)**						
(+)	1497 (19%)	414 (36%)	320 (35%)	94 (38%)	63 (33%)	351 (36%)
(−)	6241 (81%)	745 (64%)	594 (65%)	151 (62%)	130 (67%)	615 (64%)
**Univariate**						
OR	Ref	2.31	2.25	2.59	2.02	2.38
95% CI	–	2.02–2.65	1.93–2.61	1.97–3.40	1.46–2.77	2.06–2.75
*P*-value	–	< 0.01	< 0.01	< 0.01	< 0.01	< 0.01
**Multivariate**						
OR	Ref	2.05	1.98	2.33	1.55	2.16
95% CI	–	1.78–2.37	1.69–2.33	1.74–3.11	1.11–2.16	1.85–2.52
*P*-value	–	< 0.01	< 0.01	< 0.01	< 0.01	< 0.01
**χ**^**2**^**-test**	–	–	0.33		0.37	

*Ref* reference, *OR* Odds ratio, *CI* Confidence interval.

In terms of the histological subtypes, the smoking rates in patients with UPS of bone, giant cell tumor (GCT) of bone, dermatofibrosarcoma protuberans (DFSP), liposarcoma (including myxoid type, dedifferentiated type, and pleomorphic type), and UPS of soft tissue were higher than those in controls (19%, 1497/7738): 57% (13/23), 50% (6/12), 50% (11/22), 47% (67/144), and 45% (79/175), respectively. In the multivariable analyses, the ORs for UPS of bone, UPS of soft tissue, and liposarcoma were 5.71 (95% CI 2.27–14.3, *p* < 0.01), 3.04 (95% CI 2.18–4.26, *p* < 0.01), and 2.92 (95% CI 2.03–4.20, *p* < 0.01), respectively (Table 6).

**Table 6:** Smoking rates in patients ≥ 20 years of age according to histological subtypes of sarcoma.

	Controls	Cases				
		Bone sarcoma		Soft tissue sarcoma		
		UPS	GCT	DFSP	Liposarcoma	UPS
N	7738	23	12	22	144	175
**Smoking habit (%)**						
(+)	1497 (19%)	13 (57%)	6 (50%)	11 (50%)	67 (47%)	79 (45%)
(−)	6241 (81%)	10 (43%)	6 (50%)	11 (50%)	77 (53%)	96 (55%)
**Univariate**						
OR	Ref	5.42	4.17	4.17	3.63	3.43
95% CI	–	2.19–13.8	1.11–15.6	1.64–10.6	2.56–5.12	2.500–4.69
*P*-value	–	< 0.01	0.02	< 0.01	< 0.01	< 0.01
**Multivariate**						
OR	Ref	5.71	3.20	2.50	2.92	3.04
95% CI	–	2.27–14.3	0.95–10.7	1.02–6.13	2.03–4.20	2.18–4.26
*P*-value	–	< 0.01	0.06	0.04	< 0.01	< 0.01

*Ref* reference, *UPS* Undifferentiated pleomorphic sarcoma, *GCT* Giant cell tumor of bone, *DFSP* Dermatofibrosarcoma protuberans, *OR* Odds ratio, *CI* Confidence interval.

## Discussion

Using a large set of data from sarcoma patients, we found that 8% of sarcoma patients had a personal history of other cancers and 30% had a family history of cancer in a first-degree relative. In comparison to a normal population, adult sarcoma patients were much more likely to have a smoking habit (36% vs 19%, adjusted OR 2.05). We also found other potential risk factors that did not reach the pre-specified significance level. To our knowledge, this is the largest study to investigate the incidence of personal and family cancer history in sarcoma patients, as well as to compare the lifestyle habits and lifestyle-related diseases of sarcoma patients and healthy controls in Japan.

In the present study, the rate of a personal cancer history in sarcoma patients was not very high overall, but patients with angiosarcoma (n = 6) and UPS (n = 31) of soft tissue tended to have another cancer for which radiotherapy was regularly required for standard treatment. Angiosarcoma and UPS are the most common radiation-induced sarcomas^38^. In six patients with angiosarcoma, the cancer history included four breast cancer and one cervical cancer. In 31 UPS patients, the cancer history included 5 prostate cancer, 2 breast cancer, 2 cervical cancer, 2 esophageal cancer, 2 malignant lymphoma, and 2 skin squamous cell cancer, which are considered radiosensitive cancers^39–41^. However, the number of these cases was relatively few, and the association between radiotherapy and secondary sarcoma was not determined in the present study. It was also impossible to discriminate between primary and secondary sarcoma based on the pathological findings alone; however, it was still essential to consider the possibilities of sarcomas developing in patients with a history of radiotherapy for another radiosensitive cancer.

Some sarcoma patients have been reported to have a family history of cancer^10–12^. Nabi et al. reported that 35% of sarcoma patients had a first-degree relative with a history of cancer^11^. McDuffie et al. stated that 37% of soft tissue sarcoma patients had a first-degree relative with a history of cancer^10^. These rates were close to the outcomes of the present study (Table 7). The common histological types (MPNST, leiomyosarcoma, EMC, chordoma, and synovial sarcoma) in sarcoma patients with first-degree relatives with a history of cancer are well known to be associated with genetic abnormalities. MPNST was associated with *NF1* gene mutations^42^. Leiomyosarcoma was associated with the deletion of the *TP53* and *RB1* gene and *BRCA1/2* gene rearrangement^43^. EMC was correlated with *NR4A3* gene rearrangement^44^. Chordoma was associated with the T-box-family transcription factor, brachyury^45^. The *SS18-SSX* fusion gene was shown to be involved in synovial sarcoma^46^. Recent genomic studies have expanded our knowledge regarding the basis of carcinogenesis, including sarcoma; however, in most types of sarcoma, gene abnormalities (e.g., fusion genes or driver mutations) remain unclear. Lung and breast cancer were commonly reported as types of family cancer associated with sarcoma in previous reports^11,12^. However, in the present study, gastric cancer was the most common type of family cancer in both bone and soft tissue sarcoma (Tables 3 and 7). The higher incidence of gastric cancer in comparison to U.S. or European populations may be because the population of this study was entirely Japanese, an Asian population that is particularly susceptible to gastric cancer. However, the inheritance of the genes associated with gastric cancer may have contributed to the occurrence of some types of sarcoma, and the research on the gene expression or the hereditary form of gastric cancer, may lead to the discovery of a new mechanism underlying the development of sarcoma.

**Table 7:** Previous studies regarding family cancer history and histology of sarcoma and cancer type in sarcoma patients.

Author, year (Reference)	Family cancer history		Relatives	Histological type	Cancer type
	Cases (%)	Controls (%)			
McDuffie et al., 2009^10^	Soft tissue sarcoma 133/357 (37%)	380/1506 (25%)	Up to first-degree relatives	N.A	N.A
Nabi et al., 2015^11^	Bone and Soft tissue sarcoma 147/425 (35%)	53/429 (12%)	Up to first-degree relatives	1 Leiomyosarcoma 2 Spindle cell sarcoma 3 GIST	1 Breast 2 Lung 3 Colorectal
Schiavi et al., 2015^12^	Bone and Soft tissue sarcoma 113/163 (69%)	N.A	Up to third-degree relatives	1 Liposarcoma 2 Myxofibrosarcoma 3 Leiomyosarcoma	1 Lung 2 Breast 3 Prostate
Current study 2022	Bone and Soft tissue sarcoma 367/1320 (28%) Soft tissue sarcoma 311/1046 (30%)	N.A	Up to first-degree relatives	1 MPNST 2 Leiomyosarcoma 3 Chordoma 4 EMC 5 Synovial sarcoma	1 Gastric 2 Lung 3 Colorectal

*GIST* Gastrointestinal stromal tumor, *MPNST* Malignant peripheral nerve sheath tumor, *EMC* Extraskeletal myxoid chondrosarcoma, *N.A.* Not available.

A large number of carcinogens in cigarette smoke have been implicated as contributors to oncogenesis in various types of cancer^18–23,47–49^. However, there have only been a few previous reports about the association between smoking and the development of sarcoma^10–12,50–52^. Monograph 100E, which was published in 2012 by the International Agency for Research on Cancer (IARC) in the WHO, did not report a relationship between smoking and bone sarcoma, and there was no definitive conclusion on the correlation between smoking and soft tissue sarcoma^49^. In previous studies, a cohort study with a 26-year follow-up period found an association between smoking and mortality in soft tissue sarcoma patients^10^; the relative risk (RR) was 1.8 (95% CI 1.1–2.9). In 2015, smoking was reported as a potential risk factor for sarcoma with an OR of 2.67^11^ (Table 8). Our results suggested that the ORs for sarcoma in individuals with a smoking habit were almost consistent with those for breast cancer and renal cell carcinoma, with ORs of 2.02 and 2.20, respectively^48,53^ (Table 9). Furthermore, in smokers, the ORs for types of sarcoma especially UPS of bone, UPS of soft tissue, and liposarcoma were close to or higher than the risk of developing lymphoma or upper aerodigestive tract cancer, which were previously reported to be associated with smoking^19,22^. A type of thoracic sarcoma (SMARCA4-deficient type) was reported to be highly associated with smoking (78%, 11/13)^54^. In the present study, the smoking rates of patients with pleomorphic liposarcoma, UPS of bone, and DFSP were relatively high at 63% (5/8), 57% (13/23), and 50% (11/22), respectively, although the number of patients was small (Supplementary Table 2). Further studies are needed to investigate the smoking habits in patients with these sarcomas.

**Table 8:** Outcomes for the incidence of sarcomas by smoking in previous studies.

Author, year (Reference)	Study design	Type of sarcoma	Cases	Controls	Outcome (95% CI)
Zahm et al., 1989^36^	Case–control	Soft tissue sarcoma	228	1610	OR 1.8 (1.1–2.9)
Zahm et al., 1992^35^	Cohort	Soft tissue sarcoma	119	248,046	RR 1.8 (1.1–2.9)
Nabi et al., 2015^11^	Case–control	Bone and soft tissue sarcoma	425	429	OR 2.67 (1.83–3.89)
Current study, 2022	Case–control	Bone and soft tissue sarcoma	1159 (Bone 193) (Soft tissue 966)	7738	OR 2.05 (1.78–2.37) (Bone OR 1.55 (1.11–2.16)) (Soft tissue OR 2.16 (1.85–2.52))

*CI* Confidence interval, *OR* Odds ratio, *RR* Relative risk.

**Table 9:** Odds ratios for various types of cancer in smokers in previous studies.

Types of cancer	Author (Reference)	Year	Cases	Controls	OR (95% CI)
Lung cancer	Remen et al.^18^	2018	1203	1513	7.82 (4.59–13.3)
Oropharynx cancer	Anantharaman et al.^20^	2016	458	2994	6.82 (4.52–10.3)
Upper aerodigestive tract cancer	Lee et al.^22^	2013	1586	1260	3.83 (2.56–5.73)
Lymphoma (Hodgkin)	Taborelli et al.^19^	2017	188	1004	2.47 (1.25–4.87)
Renal cell carcinoma (clear cell type)	Patel et al.^53^	2015	816	n.a	2.20 (n.a.)
Breast cancer	Dianatinasab et al.^48^	2017	526	526	2.02 (1.22–3.34)
Bladder cancer	Zheng et al.^6^	2012	1886	2716	1.8 (1.4–2.2)
Colorectal cancer	Hou et al.^21^	2014	12,942	25,884	1.6 (1.3–1.9)
Gastric cancer	Praud et al.^47^	2018	10,290	26,145	1.25 (1.11–1.40)
Sarcoma	Current study	2022	1159	7738	2.05 (1.78–2.37)

*OR* Odds ratio, *CI* Confidence interval.

The present study was associated with several limitations that warrant mention. First, the case was derived from a high-volume hospital, thus, the patient characteristics may not be representative of all Japanese patients with sarcoma. However, the hospital is a national center and treats patients from all over Japan. Second, a recall bias in sarcoma patients may have been present with regard to the lifestyle habits and family cancer history because these were self-reported in a clinical setting. Patients may have reported more lifestyle risk factors than were described in the representative nationwide survey. However, smoking was shown to be related to sarcoma while alcohol was not. The contrast suggests that recall alone does not explain the observed relationship. Third, the body mass index may change with disease progression. Weight loss is often observed in patients with advanced-stage cancer. The BMI difference may be the result of cancer rather than a risk factor. Fourth, we were unable to obtain the personal or family history of cancer in control cases; thus, we could not include personal or family cancer history in the comparative analysis. However, it would be helpful to know the incidence of other cancers in Japanese sarcoma patients as a basis for future research. Fifth, in this study, 11 patients with Neurofibromatosis type 1, 2 retinoblastoma patients, and 1 hereditary multiple exostosis patient were observed. In addition, chronic lymphoedema of the lower limb due to Stewart-Treves syndrome was observed in one patient. However, the impact of these reported risk factors for sarcoma was not statistically analyzed because of the absence of these entities in the control data. Larger studies involving multivariate analyses will be required in order to newly establish risk factors for sarcoma, including inherited disorders, lymphoedema, a history of radiotherapy, or exposure to chemicals (e.g. pesticide) while working, as was previously reported to be a risk factor for sarcoma^10–16,38,55,56^.

In conclusion, we confirmed that having a first-degree relative with a history of gastric cancer was suggested to be associated with the incidence of bone and soft tissue sarcoma; however, further studies are needed to support this association as this study had no data regarding the family history of cancer in the control group. In addition, we validated that a smoking habit was associated with the incidence of sarcoma, especially UPS of bone, UPS of soft tissue, and liposarcoma, in a comparative study.

## Methods

### Data sources

To investigate the personal or family cancer history of sarcoma patients, we enrolled 1320 patients with bone and soft tissue sarcoma who were identified in the pathological database of the National Cancer Center Hospital between 2006 and 2013. We included all patients with histopathologically-confirmed primary sarcoma according to the 2013 WHO classification of Soft Tissue and Bone Tumours 4th edition^1^. In the first-visit check-up, all patients completed a questionnaire about their personal and family history of cancer, lifestyle habits, and lifestyle-related diseases.

To examine lifestyle risk factors among the 1320 patients, we compared the case data of 1159 patients (≥ 20 years of age) to 7738 controls of ≥ 20 years of age who were derived from the National Health and Nutrition Examination Survey (NHNES)^37^ conducted by the Japanese Ministry of Health, Labour and Welfare in 2014. Individual person-level data for controls were used for the analysis. The objective of this national survey was to produce a national estimate of the current health status and lifestyle habits among Japanese people in order to provide data for health policy-making. The response rate was 67% (3648 of 5432 households that received a questionnaire). The target households were all family members in 299 unit areas selected from 11,000 unit areas throughout Japan by a stratified random sampling method.

This study was approved by the Institutional Review Board of National Cancer Central (Research Number: 2017–336) in compliance with the guidelines of the Helsinki Declaration of 1964.

### Variables and analyses

To investigate the prevalence of a personal and family cancer history in 1320 sarcoma patients based on questionnaire responses, we calculated the proportions of patients who had previously had cancer, excluding any sarcoma, as patients with a personal history of cancer. A family history of cancer included having first-, second-, or third-degree relatives with any cancer. The types of cancer with which sarcoma patients and their family were diagnosed were also investigated. Sub-analyses for differences in family history were performed according to sex, disease origin (bone or soft tissue), and histological type according to the 2013 WHO classification^1^.

To examine the risk factors for sarcoma, we compared the lifestyle habits and lifestyle-related diseases of sarcoma patients to those of normal individuals in a case–control manner. Smoking, alcohol consumption, obesity, DM, HT, DL, age, and sex were examined as possible risk factors among lifestyle habits and lifestyle-related diseases in sarcoma patients of ≥ 20 years of age. Sarcoma tends to demonstrate an age-dependent or sex-related incidence, and both age and sex were considered to be essential confounding factors, and were also included in the analyses.

To define the lifestyle variables in this study, the same categories were applied to the control group. Ever smokers were defined as individuals who had ever smoked cigarettes at least once a week for a year, and former smokers were defined as individuals who had stopped for at least one year prior to the diagnosis^18–23^. A gram per day was defined as a standard measure of ethanol intake^24–26^, using equivalents of 0.8 g mL-1. The ethanol amount in one drink of beer was equivalent to approximately 12.5 g (5% ethanol), which was almost equal to the amount in 120 mL of wine (12–13% ethanol, 12 g), 90 ml of Japanese sake (15–16% ethanol, 12 g), 40 ml of spirit (30–40% ethanol, 10–12 g), and 30 ml of whisky (40–50% ethanol, 10–12 g). An alcohol drinking habit was defined as the consumption of an ethanol equivalent of ≥ 12.5 g per day. Obesity was defined as a body mass index (BMI) ≥ 25 kg/m^2^^32–34,57^. DM was defined as taking antidiabetic medicines or insulin injections^27,28^. HT was defined as taking an antihypertensive agent^29–31^. DL was defined as taking an antihyperlipidemic drug (such as anticholesteremic or antitriglyceride agents)^31,34^. Age was classified into the following four groups; 20–39, 40–59, and ≥ 60 years, and we set ≥ 60 years as a reference group for the multivariate analysis.

Fisher’s exact test and logistic regression model were used to compare the frequencies of potential risk factors in univariate and multivariate analyses, respectively. The variables examined included smoking, alcohol consumption, obesity, DM, HT, DL, age, and sex. We also performed sub-analyses taking the cases of each histological grade (high-grade or low to intermediate), disease origin (bone or soft tissue) and histological subtype according to the 2013 WHO classification^1^, and calculated distributions of risk factors were compared between histological grade (high vs. low and intermediate) and disease origin (bone vs. soft tissue) using the χ^2^-test. *P* values of < 0.01 were considered to indicate statistical significance.

All statistical analyses were performed using EZR (Saitama Medical Center, Jichi Medical University, Saitama, Japan), which is a graphical user interface for the R software program (The R Foundation for Statistical Computing, Vienna, Austria)^58^.

### Approval for human experiments

This study was approved by the Institutional Review Board of National Cancer Central (Research Number: 2017-336) in compliance with the guidelines of the Helsinki Declaration of 1964.

### Consent to participate/consent to publish

The written informed consent was acquired from all patients and/or their parents (in the case of children). All participants and/or their parents (in the case of children) in the NHNES study agreed to allow public release of their data. In addition, second utilization of their data in the NHNES study was formally approved by the Japanese Ministry of Health, Labour and Welfare.

## Supplementary Information

Supplementary Information.

## Data Availability

All data in the present study are included in this article.

## References

[1] Fletcher CD, Bridge JA, Hogendoorn PCW, Mertens F (2013). WHO Classification of Tumors of Soft Tissue and Bone.

[2] Ogura K, Higashi T, Kawai A (2017). Statistics of bone sarcoma in Japan: Report from the bone and soft tissue tumor registry in Japan. J. Orthop. Sci..

[3] Ogura K, Higashi T, Kawai A (2017). Statistics of soft-tissue sarcoma in Japan: Report from the bone and soft tissue tumor registry in Japan. J. Orthop. Sci..

[4] Zheng Y (2020). A new comprehensive colorectal cancer risk prediction model incorporating family history, personal characteristics, and environmental factors. Cancer Epidemiol. Biomark. Prev..

[5] Beebe-Dimmer JL (2017). Family history of prostate and colorectal cancer and risk of colorectal cancer in the Women's health initiative. BMC Cancer.

[6] Zheng YL (2012). Urinary bladder cancer risk factors in Egypt: A multicenter case–control study. Cancer Epidemiol. Biomark. Prev..

[7] Braithwaite D, Breast Cancer Surveillance Consortium (2018). Family history and breast cancer risk among older women in the Breast Cancer Surveillance Consortium cohort. JAMA Intern. Med..

[8] Kellerud RDR (2019). Family history of cancer and risk of paediatric and young adult's testicular cancer: A Norwegian cohort study. Br. J. Cancer..

[9] Liu X, Cai H, Yu L, Huang H, Long Z, Wang Y (2016). Prognostic significance of cancer family history for patients with gastric cancer: A single center experience from China. Oncotarget.

[10] McDuffie HH, Pahwa P, Karunanayake CP, Spinelli JJ, Dosman JA (2009). Clustering of cancer among families of cases with Hodgkin Lymphoma (HL), Multiple Myeloma (MM), Non-Hodgkin’s Lymphoma (NHL), Soft Tissue Sarcoma (STT) and control subjects. BMC Cancer.

[11] Nabi S, Kahlon P, Kuriakose P (2015). Analyzing multiple risk factors in patients with sarcomas. A case–control study. Indian J. Cancer..

[12] Schiavi A (2015). Using a family history questionnaire to identify adult patients with increased genetic risk for sarcoma. Curr. Oncol..

[13] Correa H (2016). Li–Fraumeni syndrome. J. Pediatr. Genet..

[14] Bougeard G (2015). Revisiting Li–Fraumeni syndrome from *TP53* mutation carriers. J. Clin. Oncol..

[15] Saita C (2018). Tumor development in Japanese patients with lynch syndrome. PLoS ONE.

[16] Latham A (2019). Microsatellite instability is associated with the presence of lynch syndrome pan-cancer. J. Clin. Oncol..

[17] Rekhtman N (2020). SMARCA4-deficient thoracic sarcomatoid tumors represent primarily smoking-related undifferentiated carcinomas rather than primary thoracic sarcomas. J. Thorac. Oncol..

[18] Remen T, Pintos J, Abrahamowicz M, Siemiatycki J (2018). Risk of lung cancer in relation to various metrics of smoking history: A case–control study in Montreal. BMC Cancer.

[19] Taborelli M (2017). The dose-response relationship between tobacco smoking and the risk of lymphomas: A case–control study. BMC Cancer.

[20] Anantharaman D (2016). Combined effects of smoking and HPV16 in oropharyngeal cancer. Int. J. Epidemiol..

[21] Hou L (2014). Association between smoking and deaths due to colorectal malignant carcinoma: A national population-based case-control study in China. Br. J. Cancer..

[22] Lee YA (2013). Smoking addiction and the risk of upper-aerodigestive-tract cancer in a multicenter case–control study. Int. J. Cancer..

[23] Gandini S (2008). Tobacco smoking and cancer: A meta-analysis. Int. J. Cancer..

[24] Zhao J, Stockwell T, Roemer A, Chikritzhs T (2016). Is alcohol consumption a risk factor for prostate cancer? A systematic review and meta-analysis. BMC Cancer.

[25] Bagnardi V (2015). Alcohol consumption and site-specific cancer risk: A comprehensive dose-response meta-analysis. Br. J. Cancer..

[26] Kawakita D, Matsuo K (2017). Alcohol and head and neck cancer. Cancer Metastasis Rev..

[27] Suh S, Kim KW (2019). Diabetes and cancer: Cancer should be screened in routine diabetes assessment. Diabetes Metab. J..

[28] Linkeviciute-Ulinskiene D, Patasius A, Zabuliene L, Stukas R, Smailyte G (2019). Increased risk of site-specific cancer in people with type 2 diabetes: A national cohort study. Int. J. Environ. Res. Public Health..

[29] Han H (2017). Hypertension and breast cancer risk: A systematic review and meta-analysis. Sci. Rep..

[30] Sanfilippo KM (2014). Hypertension and obesity and the risk of kidney cancer in 2 large cohorts of US men and women. Hypertension.

[31] Radišauskas R, Kuzmickienė I, Milinavičienė E, Everatt R (2016). Hypertension, serum lipids and cancer risk: A review of epidemiological evidence. Medicina (Kaunas)..

[32] Emma HA, Stephen DH (2015). Obesity and cancer: Mechanistic insights from transdisciplinary studies. Endocr. Relat. Cancer..

[33] Iyengar NM, Gucalp A, Dannenberg AJ, Hudis CA (2016). Obesity and cancer mechanisms: Tumor microenvironment and inflammation. J. Clin. Oncol..

[34] Howe LR, Subbaramaiah K, Hudis CA, Dannenberg AJ (2013). Molecular pathways: Adipose inflammation as a mediator of obesity-associated cancer. Clin. Cancer Res..

[35] Zahm SH, Heineman EF, Vaught JB (1992). Soft tissue sarcoma and tobacco use: Data from a prospective cohort study of United States veterans. Cancer Causes Contol..

[36] Zahm SH, Blair A, Homes FF, Boysen CD, Robel RJ, Fraumeni JF (1989). A case–control study of soft-tissue sarcoma. Am. J. Epidemiol..

[37] Japanese Ministry of Health, Labour and Welfare. *A Japanese National Health and Nutrition Examination Survey in 2014*. https://www.mhlw.go.jp/english, https://www.mhlw.go.jp/stf/houdou/0000106405

[38] Salminen SH, Sampo MM, Böhling TO, Tuomikoski L, Tarkkanen M, Blomqvist CP (2018). Radiation-associated sarcoma after breast cancer in a nationwide population: Increasing risk of angiosarcoma. Cancer Med..

[39] Díaz-Gavela AA, Del Cerro Peñalver E, Sanchez García S, Leonardo Guerrero L, Sanz Rosa D, Couñago Lorenzo F (2021). Breast cancer radiotherapy: What physicians need to know in the era of the precision medicine. Breast Dis..

[40] Catton CN (2017). Randomized Trial of a hypofractionated radiation regimen for the treatment of localized prostate cancer. J. Clin. Oncol..

[41] Scott JG (2017). A genome-based model for adjusting radiotherapy dose (GARD): A retrospective, cohort-based study. Lancet Oncol..

[42] Malbari F (2018). Malignant peripheral nerve sheath tumors in neurofibromatosis: Impact of family history. J. Pediatr. Hematol. Oncol..

[43] Chudasama P (2018). Integrative genomic and transcriptomic analysis of leiomyosarcoma. Nat. Commun..

[44] Brenca M (2019). NR4A3 fusion proteins trigger an axon guidance switch that marks the difference between EWSR1 and TAF15 translocated extraskeletal myxoid chondrosarcomas. J. Pathol..

[45] Sharifnia T (2019). Small-molecule targeting of brachyury transcription factor addiction in chordoma. Nat. Med..

[46] McBride MJ (2018). The SS18-SSX fusion oncoprotein hijacks BAF complex targeting and function to drive synovial sarcoma. Cancer Cell.

[47] Praud D (2018). Cigarette smoking and gastric cancer in the Stomach Cancer Pooling (StoP) Project. Eur. J. Cancer Prev..

[48] Dianatinasab M, Fararouei M, Mohammadianpanah M, Zare-Bandamiri M, Rezaianzadeh A (2017). Hair coloring, stress, and smoking increase the risk of breast cancer: A case–control study. Clin. Breast Cancer..

[49] IARC Monographs on the Evaluation of Carcinogenic Risks to Humans. *Volume 100E. Tobacco Smoking. Soft-Tissue Sarcoma*. Lyon, France, 109 (2012).

[50] Fidler MM, Frobisher C, Guha J, Wong K, Kelly J, Winter DL (2015). Long-term adverse outcomes in survivors of childhood bone sarcoma: The British Childhood Cancer Survivor Study. Br. J. Cancer..

[51] Franceschi S, Serraino D (1992). Risk factors for adult soft tissue sarcoma in northern Italy. Ann. Oncol..

[52] Zahm SH, Fraumeni JF (1997). The epidemiology of soft tissue sarcoma. Semin. Oncol..

[53] Patel NH (2015). Comparative analysis of smoking as a risk factor among renal cell carcinoma histological subtypes. J. Urol..

[54] Yoshida A (2017). Clinicopathological and molecular characterization of SMARCA4-deficient thoracic sarcomas with comparison to potentially related entities. Mod. Pathol..

[55] Harrison WD, Chandrasekar CR (2015). Stewart–Treves syndrome following idiopathic leg lymphoedema: Remember sarcoma. J. Wound Care..

[56] Hansen ES, Lander F, Lauritsen JM (2007). Time trends in cancer risk and pesticide exposure, a long-term follow-up of Danish gardeners. Scand. J. Work Environ. Health.

[57] WHO Expert Consultation (2004). Appropriate body-mass index for Asian populations and its implications for policy and intervention strategies. Lancet.

[58] Kanda Y (2013). Investigation of the freely available easy-to-use software ‘EZR’ for medical statistics. Bone Marrow Transplant..

