# Supplementary material for: Family cancer history and smoking habit associated with sarcoma in a Japanese population study
Source: Sci Rep. 2022 Oct 12;12:17129. doi: 10.1038/s41598-022-21500-0 (PMC9556776; doi:10.1038/s41598-022-21500-0)
Supplement: Supplementary file 1 — Supplementary Information. [file 41598_2022_21500_MOESM1_ESM.docx]

Supplementary Table 1. Personal and family history of cancer in sarcoma patients according to the histological type

| Supplementary Table 1. Personal and family history of cancer in sarcoma patients according to the histological type | | | | | |
| --- | --- | --- | --- | --- | --- |
| **Histological type of sarcoma (ICD10/ICD-O-3M)** | **Cases** | **Family history of cancers** | | | **Personal cancer history (%)** |
|  |  | **Up to third-degree relatives (%)** | **Up to second-degree relatives (%)** | **first-degree relatives (%)** |  |
| **Bone sarcoma(C40, C41)** | 274 | 142 (52%) | 127 (46%) | 56 (20%) | 14 (5%) |
| Osteosarcoma(9180/3) | 103 | 60 (58%) | 56 (54%) | 18 (17%) | 3 (3%) |
| Chondrosarcoma(9220/3,9222/3) | 61 | 27 (44%) | 25 (41%) | 15 (25%) | 6 (10%) |
| Ewing sarcoma(9364/3) | 36 | 13 (36%) | 10 (28%) | 2 (6%) | 0 (0%) |
| UPS(8802/3) | 24 | 12 (50%) | 12 (50%) | 8 (33%) | 2 (8%) |
| Chordoma(9370/3) | 17 | 11 (65%) | 10 (59%) | 7 (41%) | 1 (6%) |
| GCT(9250/1) | 14 | 11 (79%) | 9 (64%) | 2 (14%) | 0 (0%) |
| Others | 19 | 8 (42%) | 5 (26%) | 4 (21%) | 2 (11%) |
| **Soft tissue sarcoma(C47, C48, C49)** | 1046 | 578 (55%) | 524 (50%) | 311 (30%) | 94 (9%) |
| Atypical lipomatous tumor(8850/1) | 126 | 59 (47%) | 55 (44%) | 35 (28%) | 12 (10%) |
| Liposarcoma(8852/3, 8858/3, 8854/3) | 150 | 90 (60%) | 81 (54%) | 41 (27%) | 9 (6%) |
| (Myxoid(8852/3)) | (73) | (41 (56%)) | (37 (51%)) | (15 (21%)) | (4 (5%)) |
| (Dedifferentiated(8858/3)) | (66) | (43 (65%)) | (38 (58%)) | (25 (38%)) | (5 (8%)) |
| (Pleomorphic(8854/3)) | (11) | (6 (55%)) | (6 (55%)) | (1 (9%)) | (0 (0%)) |
| UPS(8802/3) | 180 | 99 (55%) | 91 (51%) | 60 (33%) | 31 (17%) |
| Myxofibrosarcoma(8811/3) | 81 | 41 (51%) | 36 (44%) | 24 (30%) | 4 (5%) |
| Rhabdomyosarcoma(8910/3, 8920/3, 8901/3) | 58 | 25 (43%) | 23 (40%) | 7 (12%) | 2 (3%) |
| Leiomyosarcoma(8890/3) | 57 | 36 (63%) | 32 (56%) | 26 (46%) | 7 (12%) |
| Desmoid type fibromatosis(8821/1) | 57 | 36 (63%) | 31 (54%) | 16 (28%) | 4 (7%) |
| Synovial sarcoma(9040/3,9041/3, 9043/3) | 54 | 34 (63%) | 31(57%) | 21 (39%) | 1 (2%) |
| Extraskeletal Ewing sarcoma(9364/3) | 29 | 16 (55%) | 13 (45%) | 5 (17%) | 2 (7%) |
| MPNST(9540/3) | 25 | 16 (64%) | 16 (64%) | 13 (52%) | 0 (0%) |
| ASPS(9581/3) | 25 | 10 (40%) | 7 (28%) | 3 (12%) | 0 (0%) |
| DFSP(8832/3) | 22 | 13 (59%) | 12 (55%) | 7 (32%) | 3 (14%) |
| Epithelioid sarcoma(8804/3) | 19 | 12 (63%) | 11 (58%) | 5 (26%) | 1 (5%) |
| Angiosarcoma(9120/3) | 16 | 8 (50%) | 6 (38%) | 3 (19%) | 6 (38%) |
| Clear cell sarcoma(9044/3) | 13 | 6 (46%) | 6 (46%) | 2 (15%) | 0 (0%) |
| EMC(9231/3) | 12 | 5 (42%) | 5 (42%) | 5 (42%) | 1 (8%) |
| Low-grade fibromyxoid sarcoma(8840/3) | 11 | 8 (73%) | 8 (73%) | 4 (36%) | 0 (0%) |
| Small round cell tumor, undifferentiated(8803/3) | 9 | 4 (44%) | 2 (22%) | 0 (0%) | 1 (11%) |
| Others | 102 | 60 (59%) | 58 (57%) | 34 (33%) | 10 (10%) |
| UPS : Undifferentiated pleomorphic sarcoma, GCT : Giant cell tumor of bone, MPNST : Malignant peripheral nerve sheath tumor, ASPS : Alveolar soft part sarcoma, DFSP : Dermatofibrosarcomatous protuberans, EMC : Extraskeletal myxoid chondrosarcoma, | | | | | |

Supplementary Table 2. Smoking rates in patients with sarcoma according to histological type

| Supplementary Table 2. Smoking rates in patients with sarcoma according to histological type | | |
| --- | --- | --- |
| **Histological types of sarcoma (ICD10/ICD-O-3M)** | **Cases** | **Cases with smoking habit (%)** |
| **Bone sarcoma(C40, C41)** | 193 | 63 (33%) |
| Osteosarcoma(9180/3) | 51 | 16 (31%) |
| Chondrosarcoma(9220/3,9222/3) | 59 | 13 (22%) |
| Ewing sarcoma(9364/3) | 15 | 2 (13%) |
| UPS(8802/3) | 23 | 13 (57%) |
| Chordoma(9370/3) | 17 | 6 (35%) |
| GCT(9250/1) | 12 | 6 (50%) |
| Others | 16 | 7 (44%) |
| **Soft tissue sarcoma(C47, C48, C49)** | 966 | 351 (36%) |
| Atypical lipomatous tumor(8850/1) | 125 | 49 (39%) |
| Liposarcoma(8852/3, 8858/3, 8854/3) | 144 | 67 (47%) |
| (Myxoid(8852/3)) | (70) | (34 (49%)) |
| (Dedifferentiated(8858/3)) | (66) | (28 (42%)) |
| (Pleomorphic(8854/3)) | (8) | (5 (63%)) |
| UPS(8802/3) | 175 | 79 (45%) |
| Myxofibrosarcoma(8811/3) | 81 | 19 (23%) |
| Rhabdomyosarcoma(8910/3, 8920/3, 8901/3) | 23 | 7 (30%) |
| Leiomyosarcoma(8890/3) | 56 | 18 (32%) |
| Desmoid type fibromatosis(8821/1) | 52 | 17 (33%) |
| Synovial sarcoma(9040/3,9041/3, 9043/3) | 49 | 18 (37%) |
| Extraskeletal Ewing sarcoma(9364/3) | 22 | 4 (18%) |
| MPNST(9540/3) | 25 | 2 (8%) |
| ASPS(9581/3) | 23 | 9 (39%) |
| DFSP(8832/3) | 22 | 11 (50%) |
| Epithelioid sarcoma(8804/3) | 19 | 4 (21%) |
| Angiosarcoma(9120/3) | 16 | 4 (25%) |
| Clear cell sarcoma(9044/3) | 13 | 2 (15%) |
| EMC(9231/3) | 12 | 4 (33%) |
| Low-grade fibromyxoid sarcoma(8840/3) | 9 | 2 (22%) |
| Small round cell tumor, undifferentiated(8803/3) | 6 | 1 (17%) |
| Others | 94 | 34 (36%) |
| DM : Diabetes mellitus, HT : Hypertention, DL : Dyslipidemia, UPS : Undifferentiaated pleomorphic sarcoma, GCT : Giant cell tumor of bone, MPNST: Malignant peripheral nerve sheath tumor, ASPS: Alveolar sof tissue part sarcoma, DFSP: Dermatofibrous sarcoma protuberans, EMC: Extraskeletal myxoid chondrosarcoma | | |
